# Supplementary material for: Learning to localize sounds in a highly reverberant environment: Machine-learning tracking of dolphin whistle-like sounds in a pool
Source: PLoS One. 2020 Jun 25;15(6):e0235155. doi: 10.1371/journal.pone.0235155 (PMC7316258; doi:10.1371/journal.pone.0235155)
Supplement: Attachment — Submitted filename: FINAL_reviewer_response.pdf [file pone.0235155.s001.pdf]

Responses to Editor and Reviewer comments are given in bold red.

PONE-D-19-12399
Machine Source Localization of Tursiops Truncatus Whistle-like Sounds in a Reverberant Aquatic Environment
PLOS ONE

Dear Dr. Woodward,

Thank you for submitting your manuscript to PLOS ONE. After careful consideration, we have decided that your manuscript does not meet our criteria for publication and must therefore be rejected.

I am sorry that we cannot be more positive on this occasion, but hope that you appreciate the reasons for this decision.

Yours sincerely,

Haru Matsumoto
Academic Editor
PLOS ONE

Additional Editor Comments (if provided):

Based on reviewer 1's comments and my own review, I regrettably inform you that your paper is rejected for the publication from PLOS ONE. The followings are my review.

The paper is not written clearly nor carefully. It is no doubt that the ML method is getting popularity as an alternative method for classification, but unfortunately, it is obscure and presentation is poor and confusing. There are many careless mistakes, which do not help the quality of the paper either. The reviewer 1 did not mention but some of the mistakes are:

1) The captions for Figs. 1 and 2 appear to be reversed.

We apologize and have fixed the issue.

2) The caption for Fig. 2 mentions 14 red circles, but there are only 7 of them in the pool picture (the one labeled Fig. 1). Also, the resolution of the pool picture is substandard; it's very pixel-y.

In the caption, we have made it clearer that the 14 testing locations are located at 7 unique locations over the pool surface (and at two depths).

The image is a capture from our operating IP camera video feed, which — networked with several other cameras — had limitations in resolution. Ultimately, the purpose of this image is both to exhibit our actual video feed and to show the approximate locations of the test locations and hydrophone arrays. We feel this capture is more appropriate than a photo taken from another device, but we thank the editor for his suggestion and would be amenable to replacement if he still thinks it preferable. In the meantime, we have upscaled the original image.

3) The caption of Fig. 1 mentions a "1024-bin" spectrogram, which is a meaningless description without knowing the sample rate. A rate is eventually given for the recordings, but it's not clear this is the same rate as in this synthetic example. In any case, you should specify the window type (Hamming?) for any spectrogram as well as the frequency resolution; see spectrograms in J. Acoust. Soc. Am. for examples.

We have added additional detail to the caption.

4) Describing a pseudo-sinusoid with "arcsin(m - sin(2 pi f)) / arcsin(2 pi f)" is really unclear. What does this expression represent? Since f is a variable in it, it's not frequency, and there's no "t" so it's not a function of time; what is it? If m is anything but 0, first arcsin() will potentially be complex-valued; what is the meaning of these values?

We particularly apologize for the confusion created by this explanation, and thank the editor for pointing out its inadequacy. We have rewritten and expanded it. Incidentally, this expression should not produce complex values with the new range given for parameter m.

5) The introduction mentions classification of signature whistles, though that's apparently not the classification task of this.

In this paper, “classification” is used in a machine learning sense, i.e., referring to prediction of categorical group membership. We have made this explicit where the term is used in the introduction.

6) Location errors should be in meter and in 3-D (XYZ).

All paper values have been converted or translated to SI.

“3D” localization error was previously given as “Euclidean” distance error. In the revised manuscript we also refer to this as “straight-line” error, and in our first result also use the term “3D.”

7) SPR-PHAT appears in the abstract without explanation.

A basic explanation of SRP (revised from SRP-PHAT, a subtype we found less successful) as well as references have been added, with accompanying testing.

Above may be simple mistakes but affects the quality of the paper. The most damaging one is that the authors discuss their ML work against the SPR-PHAT method but no test results from the SPR-PHAT are shown to substantiate their claim. Further, no detail steps or explanations of how ML was applied, which makes their work obscure and unrepeatable. Applying ML for localization is unique and it may become an alternative method for locating sound sources in the open ocean. My suggestions would be to have some outside person (from the authors' suggested reviewers' list) who do not know the project work with the authors and include the person as a coauthor.

An SRP method has been applied to the data.

More details about our ML features have been added.

Also, we likely failed to make it clear that most of the ML methods employed are well-established; all novel aspects of our application (primarily the choice of features) are in fact noted in the text. While it would be out of scope to provide a primer on all methods employed, we have added both academic and novice-friendly references for interested readers. We also note that most of the methods used have novice-friendly implementations available in Matlab, Python.

[Note: HTML markup is below. Please do not edit.]

Reviewers' comments:

Reviewer's Responses to Questions

Comments to the Author

1. Is the manuscript technically sound, and do the data support the conclusions?

The manuscript must describe a technically sound piece of scientific research with data that supports the conclusions. Experiments must have been conducted rigorously, with appropriate controls, replication, and sample sizes. The conclusions must be drawn appropriately based on the data presented.

Reviewer #1: No

2. Has the statistical analysis been performed appropriately and rigorously?

Reviewer #1: No

3. Have the authors made all data underlying the findings in their manuscript fully available?

The PLOS Data policy requires authors to make all data underlying the findings described in their manuscript fully available without restriction, with rare exception (please refer to the Data Availability Statement in the manuscript PDF file). The data should be provided as part of the manuscript or its supporting information, or deposited to a public repository. For example, in addition to summary statistics, the data points behind means, medians and variance measures should be available. If there are restrictions on publicly sharing data—e.g. participant privacy or use of data from a third party—those must be specified.

Reviewer #1: Yes

4. Is the manuscript presented in an intelligible fashion and written in standard English?

PLOS ONE does not copyedit accepted manuscripts, so the language in submitted articles must be clear, correct, and unambiguous. Any typographical or grammatical errors should be corrected at revision, so please note any specific errors here.

Reviewer #1: Yes

5. Review Comments to the Author

Please use the space provided to explain your answers to the questions above. You may also include additional comments for the author, including concerns about dual publication, research ethics, or publication ethics. (Please upload your review as an attachment if it exceeds 20,000 characters)

Reviewer #1: This manuscript presents a potentially useful/interesting approach to locating animals in a reverberant environment from their vocalizations. Unfortunately, the method is tested on unrealistically simplified scenario; while the approach appears to work in this case, I am doubtful about the applicability in realistic situations. Additional issues and details below.

1) The motivation for the work is not convincing. The authors discuss difficulties in attributing calls to individual animals, but most references given are for free-swimming (open ocean) environments; a very different situation. The situation is very different in a tank, which includes the complication of reverberation (yes) but is also a much more controlled environment, meaning that many of the “open ocean” problems can be addressed and controlled. For example, in a captive/tank environment, it should be relatively straight-forward to tag all animals, and acoustic localization in a tank environment given the 16 phone (4 sub-arrays of 4 phones each) configuration can probably be achieved using existing beamforming and/or TDOA methods (more below).

We have clarified that tags are not suitable in our environment (and likely other captive environments), as they are forbidden for husbandry reasons. We thank the reviewer for bringing this to our attention.

We have added extensive content about the main alternative method for sound localization in a captive environment, SRP, focusing on Thomas et al. 2002.

2) The test situation considered is wholly over-simplified. Data and “test” source locations are taken from only 14 points in the pool. In reality dolphins can be located anywhere in the pool. In reality, the data grid will need to be significantly refined and tests will need to be conducted consider any possible source location. Moreover, the “10.73 foot” error “for novel points” (line 231) is not acceptable and not an improvement over current methods. I suspect that the experimental difficulty associated with a finer grid spacing and high volumes of data will cause problems.

We have clarified that we were unable to obtain more testing data due to equipment removal — we are not trying to suggest 14 points is optimal. However, we feel our results still show promise and are worthy of description. In the revised manuscript, we more clearly recommend more training data be used in future applications.

We feel the stated unsuitability of the “10.73 foot” error indicated is taken out of context. Most of this error was dominated by error in the “Z” direction (as we show, X and Y error was not statistically different between ML and SRP methods); we add more explanation as to why such high error in this dimension should be expected, in particular given our limited training data in the Z direction, and why this error may not even matter to practical sound attribution (where often only XY localization is required for object matching in video, as in Thomas et al. 2002).

3) Another over-simplification is the use of stationary and omni-directional sources. In reality, dolphins will be moving (at ~1.5 m/s) and whistles are projected with a complex beam pattern. These two issues will likely confound proposed method.

We have added more emphasis to our admission of the need to test on real dolphin whistles. We have noted that other cross-correlation-based methods do not fail with faster-moving sources. We have added a reference that suggests the fundamental traces of bottlenose dolphin whistles, the focus of our method, are approximately omni-directional (as has been established in other dolphin species).

4) Since the purpose of this work is to introduce a method that allows location of calls in situations with multiple animals, it is also important to consider cases with multiple sources. In general, localizing a call from a single source at a time (as tested in this manuscript) is much simpler than when there are multiple simultaneous sources.

The instances of whistles overlapping in time, let alone time and frequency, in a small captive environment are rare. We feel that this omission is minor. Moreover, it is not unrealistic to suggest that whistles overlapping in time but not frequency could be separated with filtering. We note this issue is not addressed by SRP methods (as in Thomas et al.) either. That being said, we have explicitly stated the limitation and our position.

5) For the simple situations tested, existing beamforming and/or time-of-arrival methods would probably work quite well. These (e.g. the one used successfully by Thomas, Fristrup, and Tyack in their 2002 JASA paper – TFT2002) should be implemented and compared to the proposed method. I don't anticipate that the proposed method will offer much improvement. One exception may be for sources near the surface or tank walls; these can be more difficult for TDOA methods since the direct and reflected signals arrive in close succession, hence can be difficult to separate. These cases (sources near tank walls) should be experimentally tested.

SRP method analysis has been added. While we are unable to obtain more data for special cases (e.g., closer to tank walls), there remains a meaningful and we hope interesting comparison to SRP.

6) Discussion and conclusions are overreaching. For example, line 259 says that "currently, successful localization of whistles in similar environments is not greater than 70%". It isn't clear what 70% means nor where it comes from. Only 4 references are given for "current method" [29-32], and two of are in open-ocean environments. Other relevant references are omitted from the "[29-32]" reference, e.g. TFT 2002 clearly demonstrated high-precision localization in a similar environment.

More explicit comparison, specifically by evaluating a SRP method (reflecting TFT 2002), has been added.

7) The Abstract and Discussion (line 261) indicate that the "SPR-PHAT" method is the "primary alternative" method to that proposed here. These are the only place where this method is mentioned, but no reference is provided, and the method isn't discussed or tested. Other viable methods (like those used in TFT 2002) aren't considered "alternatives" while they do, in fact, provide a proven solution to the problem.

SRP method comparison has been added.

8) Experiments, methods and results are insufficiently described and could not be replicated. A few examples:
- Hydrophone positions (coordinates) are not specified
- Detailed tank and source configuration is not provided (only approximate dimensions and positions are given)

- What are “cross-correlation” features? And why does each sound have 6601 x 136 of them? Line 135
- What are “truncated Fourier transform” features? And why does each sound have 27126 x 12 of them? Line 136
- Results (shown in Fig 3) are very rough. It is difficult/impossible to see details 3D scatter plot like this. More detailed plots and stats should be provided re. the results.

Hydrophone and source positions were specified in the linked data set (https://doi.org/10.6084/m9.figshare.7956212). We have highlighted this in the text.

More explanation about “cross-correlation” features, “truncated Fourier transform” features has been added.

We have added histogram-like plots to better show the breakdown of our localization error.

6. PLOS authors have the option to publish the peer review history of their article (what does this mean?). If published, this will include your full peer review and any attached files.

If you choose “no”, your identity will remain anonymous but your review may still be made public.

Do you want your identity to be public for this peer review? For information about this choice, including consent withdrawal, please see our Privacy Policy.

Reviewer #1: No

[NOTE: If reviewer comments were submitted as an attachment file, they will be attached to this email and accessible via the submission site. Please log into your account, locate the manuscript record, and check for the action link "View Attachments". If this link does not appear, there are no attachment files to be viewed.]

- - - - -
For journal use only: PONEDEC3
