# Supplementary material for: Learning to localize sounds in a highly reverberant environment: Machine-learning tracking of dolphin whistle-like sounds in a pool
Source: PLoS One. 2020 Jun 25;15(6):e0235155. doi: 10.1371/journal.pone.0235155 (PMC7316258; doi:10.1371/journal.pone.0235155)
Supplement: Attachment — Submitted filename: final-cover-letterR2R.pdf [file pone.0235155.s003.pdf]

PONE-D-19-33956
Former title: Machine Source Localization of Tursiops truncatus Whistle-like Sounds in a Reverberant Aquatic Environment

New Proposed Title: Learning to localize sounds in a highly reverberant environment: machine-learning tracking of dolphin whistle-like sounds in a pool.

Dr. Haru Matsumoto
Editor, PLoS

Dear Dr. Matsumoto,

Please find herewith uploaded the hopefully final revision of our manuscript.

We believe our manuscript can have substantial impact on the work of our colleagues, and welcome your giving us this opportunity to try to make the best impression on them. Some colleagues are already applying extensions of our approach to aerial sound localization of birds in dense canopy geometries with great preliminary success. We believe, with you, that we should try to make the manuscript as accessible as possible to ensure its success.

In this revision we have striven to fulfil your recommendations, particularly to make the manuscript more accessible to the general population of practicing bioacousticians who are not simultaneously experts in machine learning. However, while doing so, we were mindful that we could not change anything so substantially as to invalidate the reviewers' comments and trigger the need for another round of re-review.

Therefore, the core of the manuscript stays exactly the same; the technical exposition and results figures are entirely unchanged. However to make the package more palatable: (a) we've changed the title to make the manuscript sound less intimidating, (b) we're rephrased the abstract, to explain more clearly where the novelty of our approach lies and make it more appealing to the casual reader; (c) instead of re-working the introduction, we've re-titled the old introduction "Motivation", and added an expository and hopefully didactic Introduction before that, where the problems of reverberation and multipath are clearly articulated for a general audience, (d) including a new figure illustrating propagation of multiple echoes and how they differentially change at different elements of a sensor array. We left the technical part of our conclusions undisturbed, but (e) added an additional "Outlook" section to discuss the generalization that this method may lead to. In addition we've added the additional figure showing the actual waveform of a received signal with reverberation. Since the ADC devices used for recording in the array were standard audio devices rather than laboratory ADCs, the power of the sonograms is uncalibrated – dB re: an arbitrary origin.

We hope we have somewhat succeeded, and look forward to your guidance.

Yours sincerely,

Marcelo Magnasco, on behalf of all authors
